# Do the status and empowerment of mothers predict their daughters’ reproductive outcomes?

**DOI:** 10.1186/s12884-017-1497-z

**Published:** 2017-11-08

**Authors:** Jessica D. Gipson, Dawn M. Upchurch

**Affiliations:** UCLA Fielding School of Public Health, Department of Community Health Sciences, 650 Charles E. Young Drive South, CHS 46-071, Los Angeles, CA 90095-1772 USA

## Abstract

**Background:**

Despite increased recognition of the important influences of women’s status and empowerment on social and health outcomes for women and their families, there are few investigations that examine the extent to which any gains in women’s empowerment may be transmitted intergenerationally, that is, between mothers and their daughters.

**Methods:**

This study seeks to address this gap by using data from a unique, longitudinal, and intergenerational dataset from Cebu, Philippines (1994–2009), to examine potential influences of the status of mothers on subsequent reproductive health outcomes among their daughters. Using data from 648 mother-daughter dyads, we examine a multidimensional set of women’s status and empowerment measures among the mothers to predict three outcomes among their daughters: sexual onset by 2009 (ages 25–26), use of family planning, and experience of an unintended pregnancy.

**Results:**

We find that that while some of the mothers’ characteristics and measures of empowerment and status were predictive of their daughters’ sexual initiation, these effects were not consistent across empowerment indicators, nor were there significant effects on two of the outcomes: use of family planning or occurrence of an unintended pregnancy. Older mothers (45+ years in 1994) and mothers who were considered to be “well-kept”, a locally defined measure of empowerment, were more likely to have daughters who had not engaged in sex by 2009 (ages 25–26). Daughters with higher educational levels were also more likely to delay sex, as compared to their peers. Among young women who had become sexually active, 54% reported an unintended pregnancy (mistimed or unwanted) by the age of 25–26, yet their mothers’ empowerment and status were not predictive of daughters’ reports of an unintended pregnancy.

**Conclusions:**

Overall, these findings suggest that further research is needed to explore more proximal impacts on young women’s reproductive behavior in this setting, given other related investigations on women’s empowerment and its linkages to sexual debut and educational attainment in this setting. Findings from this examination of daughters’ reproductive outcomes suggest that there are likely additional intervening mechanisms between onset on sexual activity and mistimed or unintended pregnancy that need further elaboration.

**Electronic supplementary material:**

The online version of this article (doi:10.1186/s12884-017-1497-z) contains supplementary material, which is available to authorized users.

## Background

A body of literature, mostly from the USA and other western settings, demonstrates the important influence of families on the subsequent generations’ family formation patterns and reproductive health outcomes (see, e.g., [1, 2]). A small subset of studies from lower- and middle-income countries [3–5] find that even amidst rapid social and demographic change, family of origin characteristics remain an important predictor of health and social outcomes in the subsequent generation. Relatively few investigations, however, have specifically examined how a mother’s social status and power may be transmitted, or may predict reproductive outcomes regarding her daughter. Examination of these issues is especially imperative, given the rapid changes that these generations of women have experienced, particularly with respect to educational attainment, childbearing patterns, and the availability of contraception.

Improvements in women’s education and status around the world have drastically altered the landscape of choices and opportunities that women have for themselves and when choosing if and when they will enter into a partnership and have children [6–8]. Increasingly, girls and women in many parts of the world have expanding options from which to choose beyond the traditional roles of childbearing and childrearing, particularly with respect to education and employment opportunities. While these changes may be realized at the individual level, these shifts often reflect broader societal changes that serve as a catalyst for the development of new aspirations and ideals, particularly for girls and women in deciding the extent to which, if at all, they wish to participate in childbearing and childrearing activities [9, 10].

Self-determination and having the ability to control, or at least influence, one’s significant life events are considered central to the empowerment of women and a reflection of women’s status within society [11]. Increasingly, and based on a burgeoning literature on the positive effects of improving women’s empowerment, global development efforts have identified women’s empowerment as a critical leverage point for improving social and health outcomes for women and their families [12]. Examination of the extent to which women’s status or empowerment may be transmitted intergenerationally has been rather limited, however, despite the substantial gains that women have achieved globally in recent decades.

Using a unique, intergenerational, and longitudinal dataset from Cebu, Philippines, we explore the relationships between the status and empowerment of mothers and the sexual and reproductive behavior of their daughters. Previous analyses of this population found significant influences of mothers’ empowerment and status on children’s educational attainment [13, 14]. This study seeks to explore the potential extension of these mechanisms by examining daughters’ reproductive outcomes. Specifically, we use longitudinal data from mothers and their daughters (1994–2009) to examine reproductive outcomes among the daughters, including their sexual and contraceptive experience, as well as their experience of an unintended pregnancy by 2009, when the daughters were 25–26 years of age.

### The Philippines

The setting for this study is Metro Cebu, the second largest metropolitan area of the Philippines, located on the island of Cebu in the Central Visayas region. The Philippines has undergone substantial changes over the past few decades, namely with respect to urbanization, a shift towards nuclear versus extended households, increased usage of mobile and Internet technology, and expanded employment and educational opportunities, especially in metropolitan areas such as Cebu [15].

These changes in the social context are reflected in family formation and childbearing patterns, as well. Filipinos are now more likely to delay marriage, to choose cohabitation over formal marriage, and to engage in premarital sex than in previous generations [16–18]. Younger generations are also choosing to have smaller families — fertility has declined drastically in the Philippines over the past 40 years, from a total fertility rate of 6.0 in 1973 to 3.0 in 2013 [19]. These declines in fertility, however, have slowed over the past 10–15 years amidst controversy regarding the public provision of contraception and contraceptive prevalence rates that lag behind those of neighboring countries, with 55% of currently married women reporting use of contraception (a large proportion consisting of traditional method use), and 18% of women reporting an unmet need for family planning [19, 20]. Moreover, nearly three in ten births are considered to be unintended — 11% were reported as not wanted at all, and 17% reported as wanted later [19].

Despite the ongoing challenges in the reproductive health realm, the situation and status of women in the Philippines are notable. In comparison with other Asian countries and with lower- and middle-income settings, the women in the Philippines have historically had a higher degree of societal status and power [21]. Women compare favorably to men in terms of literacy, enrollment and completion of primary and secondary school, as well as in the proportion of women to men holding supervisory positions in labor and government sectors [22, 23]. Filipino women often make household financial decisions [24]. Some evidence suggests, however, that wives may attribute the household decision-making to their husbands even if the wives actually make the decisions [25], and another study suggests that husbands may ultimately have greater say in deciding how household earnings are allocated [26].

Although Filipino women engage in relatively egalitarian decision-making patterns in their relationships, their power is considered to be much more constrained within the private domain, that is, decisions regarding marriage and reproduction [9]. Within this realm, women may feel more pressure to adhere to traditional feminine norms indicating that a woman should remain *mahinhin,* or modest about expressing sexual desire or interest [17]. This pattern of inconsistency across decision-making domains has been noted in other settings, as well, with studies suggesting that traditionally patriarchal practices such as childbearing may be more resistant to change as compared to economic empowerment and education [10, 11].

Several studies conducted previously in Cebu attest to the importance of women’s status and empowerment in social and health outcomes for women themselves, as well as their children.

In these studies, joint marital/parental household decision-making was found to be associated with a decreased risk of violence for women [27], fewer reports of violence among children [4], and higher educational attainment among daughters. Another study found that women’s household decision-making autonomy (i.e., having final say on decisions) was associated with longer birth spacing [28]. While most studies find a protective effect of joint household decision-making, two additional studies indicate that domination of household decision-making by either partner and women’s higher relationship power were associated with higher levels of intimate partner violence [27, 29].

In studies conducted in the Philippines focusing on women’s empowerment and intergenerational effects on their children’s sexual behavior, joint parental decision-making and mother’s higher educational aspirations were associated with delayed first sex among boys, whereas the mother’s higher status (e.g., higher education) was associated with delayed sexual initiation among their daughters [30]. These studies underscore the importance of examining the intergenerational effects of women’s empowerment on children’s reproductive behavior, yet to date examinations of outcomes beyond sexual debut have not been investigated.

The current study expands upon this earlier work by explicitly examining the extent to which mothers’ empowerment and status are transferred to their daughters as manifested by their reproductive behaviors. In particular, our focus is on the effects that a mother’s status and empowerment may have on her daughter’s use of contraception and experience of unintended pregnancy. Our conceptual model incorporates a culturally tailored life-course perspective that considers women’s reproductive choices and behaviors to be a function of both family and individual dimensions [31]. We propose that young women’s reproductive attitudes and behaviors are shaped by interpersonal relationships, early life experiences, and the social status of their family of origin. We are particularly interested in examining how mothers’ levels of status and empowerment, as measured by a multidimensional set of measures, may be transmitted to their daughters during the critical life stage of adolescence (age 15), and how these influences may endure and manifest vis-a-vis daughters’ reproductive outcomes (by age 25–26). We hypothesize that, as compared to their peers, daughters of mothers with higher levels of status and empowerment will be more likely to delay sex, will have a greater likelihood of ever using contraception, and will be less likely to have experienced an unintended pregnancy. In addition, our model includes characteristics of the young women, with an emphasis on assessing the effects of the daughter’s own status (operationalized as her educational attainment) on reproductive behaviors.

## Methods

Intergenerational, longitudinal data are analyzed from mother-daughter dyads participating in the Cebu Longitudinal Health and Nutrition Survey (CLHNS) in Cebu, Philippines from 1994 to 2009. The CLHNS is a longitudinal study of Filipino mothers and their children born in 1983–1984. Mothers were selected from 33 communities (*barangays*) in Metro Cebu, the second largest metropolitan area in the Philippines. Further details of the study are described elsewhere [32, 33]. The Office of Population Studies granted permission for access to and use of the data for analysis.

This analysis uses data on women’s status and empowerment collected from mothers in the 1994 CLHNS survey and 1998 data collected from the daughters (singleton female index children (ICs); approximately 15 years old) to predict subsequent sexual and reproductive outcomes reported in the 2009 CLHNS survey when the daughters were 25–26 years old. The analytic sample comprises cohabiting mother-daughter dyads in which the mothers were married or cohabiting with the IC’s father in 1994 and daughters for whom there were educational and pregnancy history data in 2009. The sample then excluded respondents who had missing data on key variables, resulting in a final analytic sample of 648 mother-daughter dyads.

Outcome variables consist of three main variables: if the daughter reported having sex by 2009, if the daughter reported ever using contraception (modern or traditional) by 2009, and if the daughter reported ever experiencing an unintended pregnancy by 2009. Unintended pregnancy is a constructed measure based on the adult daughter’s pregnancy history and her reported response to the question: At the time you were pregnant with [X] pregnancy, did you want to become pregnant then, did you want to wait until later, or did you want no (no more) children at all? Women who reported that they wanted to wait until later were characterized as having a mistimed pregnancy, whereas those who reported that they did not want any more children were classified as having an unwanted pregnancy. For the purpose of the analysis and concern regarding smaller sample sizes for the unwanted category, we combined these categories to identify those pregnancies that were considered unintended (either mistimed or unwanted).

### Women’s status and empowerment measures

The sociodemographic and women’s status and empowerment measures were abstracted from the 1994 mothers’ survey. In addition to sociodemographic characteristics which can also serve as proxies of women’s status in society (i.e., mother’s age, education, number of living children), there were five measures of women’s empowerment and status: household decision-making, being “well-kept”, having control over money, and spousal age and educational differences. Joint decision-making was a sum scale including the number of household decisions that women made jointly with their spouses (e.g., children’s schooling, buying children’s clothes, and making major household purchases) [14, 27]. The well-kept measure was identified in a previous qualitative study in Cebu as a contextually relevant measure of women’s status [34] and has been subsequently validated and demonstrated as empirically relevant in other analyses of this population [35]. In the 1994 mother’s survey, interviewers were asked to discreetly observe the woman and her immediate household environment to determine if they thought the woman, her household, and her children were found to be “neat and tidy” [34]. The third variable was constructed based on women’s reports that their husbands turned over all of their money to them, a locally relevant indicator of household decision-making and financial management. Finally, based on previous findings indicating that spousal age and/or educational difference is related to women’s empowerment and household decision-making in the Philippines [36] and in Cebu [35], variables were constructed to reflect differences in age and educational attainment of the husband and wife.

### Covariates

Household characteristics (urban/rural) and household wealth were also included. Household wealth was based on the number of selected items in the household (e.g., color television, refrigerator, bicycle, electricity) (12 items; Cronbach’s α = 0.80; mean = 4.0) and was categorized into low (0–2 items), medium (3–5 items), and high (6 or more items).

Two measures were also abstracted from the 1998 survey of the daughters (female ICs) that serve as measures of the interaction and closeness between the mother and her daughter. The 1998 survey was the first survey in which the ICs were asked to fill out the survey independent from their mothers. Thus, we include the data here as the first direct reports of the mother-daughter interaction from the daughter’s own perspective. The first is a measure of reported closeness, as reported by the daughter in the 1998 survey. A second measure was the level of reported communication between mother and daughter on up to six topics: friends, crushes, dating, menstruation, boyfriend, and marriage. We also include a measure of the daughter’s completed education by 2009.

### Analyses

In order to more fully capture the sexual and reproductive health experiences of the daughters through 2009, we first divided the sample into three subgroups according to their sexual and reproductive experiences. The first group consists of daughters who did not have sex by 2009 (*n* = 136), the second group consists of daughters who had had sex but who had never used contraception (modern or traditional) by 2009 (*n* = 113), and the last group consists of daughters who had had sex but who had ever used contraception (*n* = 399).

We first described the characteristics of the overall sample and of these subgroups, as well as test for differences between the subgroups using analysis of variance (ANOVA), chi-square, and Bonferroni tests. We then conducted bivariate and multivariate multinomial logistic regression to determine which factors are associated with a greater likelihood of being in either the second or the third group, as compared to the first (did not have sex by 2009). We then conducted backward stepwise regressions to identify and report on the most parsimonious, data-driven models.

Next, we conducted bivariate and multivariate logistic regressions to assess the odds of the daughter experiencing an unintended pregnancy. Due to collinearity between the daughter’s education and her reported number of live births, education was removed from the final models since it was not found to be significantly associated with experience of an unintended pregnancy in the bivariate models. Full, multivariate models were estimated; however, there were no significant effects found for the women’s empowerment variables other than the mother’s reported parity in 1994. Additionally, only the daughter’s parity and ever use of family planning were found to be predictive in these models; thus, simplified models are presented.

## Results

A total of 512 of the daughters reported having sex by 2009 (ages 25–26) (79% of the sample). The mean age at first sex was 19.61 (median = 19), though the range was from 13 to 25 years. Of those who had had sex, 78% reported using family planning. Among those who reported ever use of family planning, the predominant methods reported were withdrawal, the pill, and rhythm.

Table 1 summarizes the characteristics of the three analytic subgroups: daughters who did not have sex by 2009 (*n* = 136); daughters who had sex but had not ever used contraception by 2009 (*n* = 113); daughters who had sex and had used any form of contraception by 2009 (*n* = 399). There were statistically significant differences between the groups; namely, mother’s education, the proportion reported as well-kept, and household wealth were all higher for daughters who had not had sex by 2009 as compared to the other groups.

**Table 1:** Distribution of variables for mother-daughter dyads (1994–2009), *n* = 648

Characteristics	Total	Daughter did not have sex by 2009	Daughter had sex but has not ever used contraception	Daughter had sex and has used contraception	*F* test/chi-squared test
Daughter’s sex and contraceptive experience, 2009		136	113	399	
Percent of full sample		21%	17%	62%	
Percent of sexually active sample (*n* = 512)		–	22%	78%	
Mother’s status and empowerment					
Mother’s age, mean years (SD, range)	38.10 (5.91, 26–57)	38.95	37.82	37.88	0.17
Mother’s education, mean years completed (SD, range)	7.19 (3.70, 0–17)	8.11	6.73**	7.01**	0.004
Number of living children (1994), mean (SD, range)	5.01 (2.20, 1–13)	4.63	5.13	5.11†	0.08
Number of joint household decisions (SD, range)	4.63 (2.48, 0–10)	4.72	4.66	4.59	0.87
Mother, child, and household are well-kept: at least one reported	41%	53%	34%**	39%*	0.003
Father gives all earnings to mother	74%	76%	76%	73%	0.67
Age difference (mother-father)	–2.37 (4.28, –31 to 12)	–2.04	–2.64	–2.4	0.54
Educational difference (mother-father)	–0.36 (3.30, –11 to 11)	-0.04	–0.44	–0.45	0.45
Household and father characteristics					
Father’s age, mean years (SD, range)	40.46 (6.71, 25–67)	40.99	40.46	40.29	0.57
Father’s education, mean years completed (SD, range)	7.55 (3.85, 0–19)	8.15	7.17	7.46	0.10
Urban residence	70%	68%	65%	73%	0.19
Household wealth, mean items (SD, range)	3.98 (2.89, 0–11)	4.47	3.54*	3.94	0.04
Index child (1998 and 2009)					
Reported not being close to mother (1998)	83%	86%	85%	81%	0.39
Reported discussing 3+ topics with mother (1998)	24%	21%	28%	24%	0.44
Completed education (2009)	11.49 (2.87, 2–17)	12.59	10.79***	11.32***	<0.0001

Bonferroni tests with “daughter did not have sex by 2009” as reference group: ***p ≤ 0.001, **p ≤ 0.01, *p ≤ 0.05, †*p* ≤ 0.10

*SD* standard deviation

Table 2 depicts the results of the subanalysis of women who had had at least one pregnancy by 2009 (*n* = 402). The mean number of pregnancies and live births were 2.07 and 1.72, respectively (range 1–8; range 0–7). Among this group of women, 54% reported having at least one unintended pregnancy by the 2009 survey, or 25–26 years of age. Of women reporting an unintended pregnancy (*n* = 216), approximately 10% reported more than one unintended pregnancy (*n* = 22), 75% of women reported one mistimed pregnancy, and the remaining 15% of women reported one unwanted pregnancy.

**Table 2:** Fertility characteristics of daughters who had 1+ pregnancies by 2009 (*n* = 402)

Characteristics	Total
Mean number of pregnancies	2.07
Mean number of live births	1.72
Percent reporting at least one unintended pregnancy	54%
Percent reporting a mistimed pregnancy only (*n* = 161)	40%
Percent reporting an unwanted pregnancy only (*n* = 33)	8.2%
Percent reporting both a mistimed and an unwanted pregnancy	5.5%

Table 3 depicts the results from the multinomial logistic regression predicting the daughter’s sexual and contraceptive experience. The adjusted models control for all of the variables indicated in Table 3 — women’s status and empowerment variables, household characteristics, and reports from the daughter regarding her education (as of 2009) and closeness and communication with her mother (reported in 1998). Daughters with at least a secondary education were significantly less likely to have been sexually active, either with or without reported use of contraception by 2009. Daughters with post-secondary versus elementary education were particularly less likely to have reported having sex, either with past use of contraception (Adjusted relative risk (ARR) = 0.20; *p*-value: 0.016) or without past use of contraception (ARR=0.09; *p*-value: 0.001), as compared to not having sex by 2009. Daughters’ and mothers’ measure of being well-kept was highly predictive of daughters’ sexual and contraceptive subgroup in the bivariate models, though in the multivariate model these effects only persisted for daughters who reported having had sex but not using contraception by 2009, as compared to daughters who reported not having sex (ARR = 0.55; *p* value = 0.05). Mother’s age was also predictive, with mothers who were 45+ years versus < 35 years in 1994 being significantly less likely to have daughters who were in the “had sex/ever use of contraception” group versus the no sex group (ARR = 0.41; *p* value = 0.01). Mother’s parity was only marginally significant for the latter subgroup.

**Table 3:** Bivariate and multivariate multinomial logistic models predicting daughter’s sexual and contraceptive experience, in relation to daughter having no sexual experience by 2009 (*n* = 648)

	Daughter had sex and has not used contraception				Daughter had sex and has used contraception			
	Unadjusted relative risk		Adjusted relative risk		Unadjusted relative risk		Adjusted relative risk	
	Unadjusted	95% CI	Adjusted	95% CI	Unadjusted	95% CI	Adjusted	95% CI
Mother’s status and empowerment								
Mother’s age: < 35 years (ref)	1.00	–	1.00	–	1.00	–	1.00	–
35–44 years	0.61†	(0.34–1.08)	0.63	0.34–1.18	0.69	(0.43–1.10)	0.63†	0.34–1.18
45+ years	0.52†	(0.24–1.11)	0.49	0.21–1.18	0.53*	(0.29–0.96)	0.41**	0.21–0.81
Mother’s education: ≤ eElementary (ref)	1.00	–	1.00	–	1.00	–	1.00	–
> eElementary	0.58*	(0.35–0.97)	1.04	0.55–1.98	0.70†	(0.48–1.04)	0.97	0.59–1.60
Number of living children (1994)	1.11†	(0.99–1.26)	1.05	0.91–1.21	1.11*	(1.01–1.22)	1.11†	0.99–1.24
Number of joint household decisions	0.99	(0.90–1.10)	0.98	0.88–1.09	0.98	(0.91–1.06)	0.98	0.90–1.07
Mother, child, and household are well-kept	0.45**	(0.27–0.75)	0.55*	0.31–1.00	0.56**	(0.38–0.84)	0.72	0.46–1.13
Father gives all earnings to mother	0.98	(0.55–1.76)	0.92	0.49–1.71	0.84	(0.53–1.32)	0.83	0.51–1.34
Age difference: Father ≤ 5 years older (ref)	1.00	–	1.00	–	1.00	–	1.00	–
Father > 5 years older	1.09	(0.53–2.22)	1.05	0.50–2.21	1.36	(0.78–2.40)	1.30	0.72–2.34
Mother is same age or older	0.81	(0.46–1.42)	0.95	0.51–1.78	1.18	(0.76–1.82)	1.49	0.92–2.40
Educational difference: Mother/father have equal education (+/– 1 year; ref)	1.00	–	1.00	–	1.00	–	1.00	–
Father has ≥ 2 years	1.05	(0.58–1.90)	1.16	0.62–2.16	0.99	(0.62–1.60)	1.05	0.64–1.74
Mother has ≥ 2 years	0.59†	(0.31–1.11)	0.62	0.31–1.22	0.65†	(0.40–1.05)	0.72	0.43–1.20
Household characteristics^a^								
Residence: Rural (ref)	1.00	–	1.00	–	1.00	–	1.00	–
Urban	0.87	(0.52–1.48)	1.04	0.57–1.89	1.27	(0.83–1.94)	1.47	0.91–2.38
Household wealth: Low (0–2 items) (ref)	1.00	–	1.00	–	1.00	–	1.00	–
Medium 3–5 items	0.71	(0.38–1.31)	0.97	0.49–1.90	0.91	(0.56–1.48)	1.04	0.61–1.78
High 6–12 items	0.52*	(0.28–0.95)	1.25	0.57–2.73	0.64†	(0.40–1.02)	1.02	0.55–1.86
Index child (1998 and 2009)								
Reported not being close to mother (1998)	1.09	(0.54–2.21)	1.06	0.50-2.24	1.40	(0.81–2.42)	1.25	0.77–2.10
Reported discussing 3+ topics with mother (1998)	1.46	(0.82–2.60)	1.62	0.87-3.02	1.19	(0.74–1.90)	1.27	0.77–2.10
Completed education (2009): Elementary (ref)	1.00	–	1.00	–	1.00	–	1.00	–
Secondary	0.20*	(0.06–0.73)	0.24*	0.06–0.90	0.31†	(0.09–1.04)	0.35	0.10–1.23
Post-secondary	0.08***	(0.02–0.29)	0.09***	0.02–0.39	0.17**	(0.05–0.56)	0.20*	0.06–0.74

***p ≤ 0.001, **p ≤ 0.01, *p ≤ 0.05, †*p* ≤ 0.10

^a^Father characteristics for this table are included in the age and educational difference variables in Mother’s status and empowerment

*CI* confidence interval

As noted in Table 4, the bivariate models predicting daughter’s experience of an unintended pregnancy did not indicate any significant relationships with the mother’s status and empowerment variables. The most important predictor of daughter’s report of an unintended pregnancy was parity/reported number of live births. Further exploration of the data indicated that the relationships between the covariates varied according to mistimed versus unwanted births. Moreover, the number of live births and the daughter’s completed education were highly correlated (–0.45). As a result, the multivariate models in Table 5 control for the women’s status and empowerment variables, yet they highlight the key, significant predictors after omitting the daughter’s education. As noted, the mother’s parity and the daughter’s ever use of family planning were predictive of lower odds of an unwanted pregnancy (odds ratio (OR) = 0.83; *p* value = 0.03; OR = 0.41; *p* value = 0.03, respectively), while the daughter’s parity was highly associated with her report of an unwanted pregnancy (OR = 1.82; *p* value ≤ 0.001). For mistimed pregnancy, only the daughter’s parity was significantly associated (OR = 1.55; *p* value ≤ 0.001).

**Table 4:** Bivariate odds ratios (ORs) of daughter experiencing unintended pregnancy by 2009 (*n* = 402)

	Bivariate ORs	95% confidence interval (CI)
Mother’s status and empowerment		
Mother’s age: < 35 years (ref)	1.00	–
35–44 years	0.84	0.54-1.30
45+ years	0.72	0.39–1.35
Number of joint household decisions	0.93	(0.86–1.01)
Mother, child, and household are well-kept	0.91	0.60–1.38
Father gives all earnings to mother	1.01	0.65–1.59
Age difference: Father ≤ 5 years older (ref)	1.00	–
Father > 5 years older	0.68	(0.39–1.17)
Mother is same age or older	0.79	(0.51–1.22)
Educational difference: Mother/father have equal education (+/– 1 year; ref)	1.00	–
Father has ≥ 2 years	1.02	(0.65–1.62)
Mother has ≥ 2 years	0.97	(0.59–1.58)
Household characteristics		
Residence: Rural (ref)	1.00	–
Urban	0.95	0.63-1.45
Household wealth: Low (0–2 items) (ref)	1.00	–
Medium 3–5 items	0.85	0.54–1.33
High 6–12 items	0.75	0.45–1.25
Index child (1998 and 2009)		
Reported not being close to mother (1998)	1.53	0.90–2.60
Reported discussing 3+ topics with mother (1998)	1.23	0.77–1.96
Completed education (2009): Elementary (ref)	1.00	–
Secondary	0.92	0.49–1.74
Post-secondary	0.78	0.39–1.58
Number of live births	1.66***	1.35–2.03
Ever used family planning	0.93	0.55–1.58

***p ≤ 0.001

**Table 5:** Adjusted odds of daughter experiencing a mistimed or an unwanted pregnancy (*n* = 402)

	Mistimed	95% CI	Unwanted	95% CI
Mother’s number of living children (1994)	1.01	(0.91–1.13)	0.83*	(0.70–0.99)
Daughter’s number of live births	1.55***	(1.26–1.90)	1.82^***^	(1.40–2.37)
Daughter ever used family planning	0.93	(0.18–0.93)	0.41*	(0.53–1.64)

***p ≤ 0.001; * p ≤ 0.05

Models adjusted for all women’s status and empowerment variables, household, and IC characteristics reported in previous tables. Daughter’s education (2009) was not included due to collinearity with number of live births

*CI* confidence interval

## Discussion

Findings from this sample of mother-daughter dyads indicate that while some of the mothers’ characteristics and measures of empowerment and status were predictive of their daughters’ sexual initiation, these effects were not consistent across empowerment indicators, nor were there significant effects on two of the outcomes: use of family planning or occurrence of an unintended pregnancy.

Similar to past findings from this population, several indicators of the mother’s status were predictive of her daughter’s sexual behavior. Older mothers (45+ years in 1994) and mothers who were considered to be “well-kept” were more likely to have daughters who had not engaged in sex by 2009 (ages 25–26). Further examination of the well-kept measures confirmed that these measures are not highly correlated with socioeconomic status (SES)/wealth (0.39) or mother’s education (0.30), and demonstrated independent, significant effects even in the multivariable models. This well-kept measure in this and other analyses appears to be capturing an additional dimension of women’s well-being and empowerment that is not captured by parity, SES, wealth, or any of the additional proxy measures used in analysis (and broadly used in the field) (e.g., age and educational differences, household decision-making, etc.) [35]. As might be expected due to the strong relationship between women’s education and their sexual/childbearing status, daughters with higher educational levels were also more likely to delay sex. In a further analysis to assess whether daughters’ education was associated with reports of ever using contraception, daughters who had reported ever use of contraception reported marginally higher levels of education than daughters who had never used contraception (11.3 versus 10.8 years, *p* value 0.09), though no other indicators of the mother’s status or empowerment were significantly associated with contraceptive use among their daughters. To further examine the role of daughters’ education in the pathway, we compared models with and without daughters’ education; however, there are no substantial differences in the effects sizes or significance of key variables indicating persistent, direct effects of selected mothers’ status and empowerment variables on initiation of first sex.

Although measures of mothers’ status and empowerment status have been found to exert important intergenerational influences on their sons' and daughters' timing of sexual initiation and number of children [3, 30, 37], it appears from this analysis that these effects do not extend to contraceptive use or to the occurrence of an unintended pregnancy. These null findings demonstrate the need to examine more proximal factors that influence the occurrence of an unintended pregnancy among these young women. Previous studies from the Philippines point to other areas of influence on women's reproductive and contraceptive behavior, namely ongoing restrictions in the provision of reproductive health information and supplies [38], as well as the substantial influence that male partners wield on contraceptive decision-making, as reasons for contraceptive unmet need among women [39, 40].

Further exploration of the predictors and possible interventions to address unintended pregnancy in this population are clearly needed, however, given that 54% of the women in this sample reported at least one unintended pregnancy by the age of 25–26. The majority of unintended pregnancies were reported by young women in this sample as “mistimed” or wanted 2 or more years later. It was not possible to determine the timing of these reports with respect to the woman’s lifecourse and parity; however, national-level data from the Philippines indicates a steep increase in premarital sex and a doubling of the proportion of young women (ages 15–19) who have begun childbearing — from 6.3% in 2002 to 13.6% in 2013 [41]. These trends underscore the need for improved contraceptive access and education to assist young women in either postponing or spacing pregnancies until their desired time, an issue that has continued to be challenged in the Philippines, even in the wake of the passage of the Reproductive Health Bill in 2012 [42]. This high level of unintended pregnancy (and the proportion reported as “unwanted”) is also noteworthy given the illegality of abortion in this setting. Although some women may be more likely to carry unwanted pregnancies to term amidst these legal restrictions, there is also evidence from this setting indicating that women will take matters into their own hands, and may resort to unsafe means to end unwanted pregnancies [43].

Given the high level of unintended pregnancy in this relatively young cohort of women, it is also notable that while more than one-quarter reported using family planning, many relied on traditional and less-effective methods of contraception (withdrawal and rhythm). These findings, in combination with complementary findings on these contraceptive patterns in national-level data, suggest that efforts to address unintended pregnancy might best be focused on expanding knowledge and use of a broader range of contraceptive methods, including more effective, modern methods. In addition, qualitative data from this setting indicate that contraceptive methods, particularly modern ones, may not be well known, are not readily available, and may not even be even considered (particularly by young adults) until after one or more children are born or desired family size is achieved [44, 45].

Finally, examination of the predictors of contraceptive use and of unintended pregnancy in this population highlights the need to examine the complex and multiple pathways that may influence these outcomes independently. One example of this is the finding that while the daughter’s education was marginally associated with contraceptive use, there was no significant relationship between her education and occurrence of an unintended pregnancy. This disconnect could speak to a variety of mechanisms, including incorrect use or use of an ineffective contraceptive method, as well as differences in family size preferences among subsets of the population that will affect their characterizations of a pregnancy as unintended (either mistimed or unwanted). Further investigations are needed that explore these pathways as they pertain to women’s status and empowerment, particularly in better ensuring that women are achieving their childbearing desires.

A few limitations should be noted from this analysis. Although the sample was of sufficient size to test these mechanisms, the subsample for this analysis was also limited to daughters for whom we had longitudinal data available, both for them and for their mothers and fathers, thereby increasing the selectivity of the sample. This limitation is balanced, however, by the ability to examine these influences over time and for both mothers and fathers. A second caveat to the exploration of unintended pregnancy in this sample is that, similar to many other data sources (e.g., Demographic and Health Surveys), information on pregnancy intention was ascertained retrospectively on reported pregnancies. This method of data collection is likely to underreport unintended pregnancies, as well as pregnancies that were terminated, thereby underestimating the true level of unintended pregnancy in the population.

## Conclusions

Overall, this study finds mixed effects of measures of maternal status and empowerment on daughters’ sexual debut and reproductive outcomes. Although there was some evidence that key indicators of women’s empowerment were predictive of their daughters’ sexual initiation, these effects were not consistent. Among young women who have become sexually active, however, women’s empowerment was not predictive of mistimed or unintended pregnancy. These findings suggest that further research is needed to explore more proximal impacts on young women’s reproductive behavior in this setting. There may be additional intervening mechanisms between onset of sexual activity and mistimed or unintended pregnancy that need further elaboration.

### Open peer review

Peer review reports for this article are available in Additional file 1.

## Additional file

Additional file 1: Open peer review. (PDF 166 kb)
