# Supplementary material for: Do the status and empowerment of mothers predict their daughters’ reproductive outcomes?
Source: BMC Pregnancy Childbirth. 2017 Nov 8;17(Suppl 2):348. doi: 10.1186/s12884-017-1497-z (PMC5688505; doi:10.1186/s12884-017-1497-z)
Supplement: Additional file 1: — Open peer review. (PDF 166 kb) [file 12884_2017_1497_MOESM1_ESM.pdf]

Review reports

Title: Does mothers' status and empowerment predict daughters' reproductive outcomes?

Reviewer 1: Caitlin Gerdts

This is a beautifully written paper with a well conducted analysis that explores a relevant topic within a unique dataset, and will make an important contribution to the field. There are a few outstanding issues that the authors should address before the paper is ready for publication.

Major Compulsory Revisions

1. The empowerment "exposure variables" along with the daughters measures of closeness/communication were abstracted from the 1994 and 1998 surveys, which were collected 15 and 11 years (respectively) prior to the outcome measures. While there is an argument to be made that the mothers level of empowerment at that time has a predictive effect on the outcomes of interest, there is another argument to be made that empowerment levels, feelings of closeness, and even some of the basic socio-demographic characteristics that are included as covariates may change over time and those changed levels of exposures/covariates of interest (closer in time to the measured outcomes) may have more of an impact on the outcomes of interest than the levels measured in 1994/1998. It is possible that the authors do not have access to these same covariates at any other point in time, in which case they should discuss the relative merits and challenges with that lack of data and likely include that discussion in the limitations section. Or they may have access to such data and have made a conceptual decision not to include time varying covariates in the analysis. Either way, the authors must engage in a discussion of why the current conceptual model that measures levels of empowerment/closeness/communication in 1994/1998 is a reasonable model with which to predict the outcomes of interest 11-15 years later without accounting for changes in the exposure variables.

2. Paragraph 2 of the Results section. It would be very helpful to see these data presented in tabular form.

3. Paragraph 4 of the Results section (beginning: Table 2…) needs to describe the difference between the adjusted and unadjusted models. What was controlled for exactly in the adjusted models?

4. Paragraph 5 of the Results section, lines 1-5, belong in the methods section, and I would like to see a more robust explanation of the theoretical construction of the model (this inevitably relates to comment #1)

5. Paragraph 5 of the results section: I am not sure that you gain anything from the inclusion of the backwards stepwise model in the text. The "data driven model" approach can be useful at times, but given that you have some issues of selectivity in your sample, and may be missing some time varying factors, I would encourage you to stick with the conceptual/theoretically driven model as it has stronger a priori grounding and doesn't rely on data (which might be biased) to drive the model.

6. Paragraph 4 of the discussion section: you note that the rate of unintended pregnancy is alarmingly high in this population, but that is combining mistimed (75% of the sample) and unwanted pregnancies (15% of the sample). It seems like a bit more nuanced discussion of the potential differences between the two is warranted.

Level of interest - An article of importance in its field

Quality of written English - Acceptable

I declare that I have no competing interests

Reviewer 2: Julianna Deardorff

I appreciated the opportunity to read this manuscript titled "Does mothers' status and empowerment predict daughters' reproductive outcomes?" which examined associations between maternal status/empowerment and daughters' sexual and reproductive outcomes. This study is interesting in its examination of intergenerational transmission of maternal status to reproductive outcomes in the next generation of girls and women using longitudinal data from the Philippines. Results showed that higher maternal status and education (not necessarily empowerment) was associated with one outcome, delayed sex among daughters, but not the other outcomes, contraceptive use or unintended pregnancy. The manuscript is well-written, the area of investigation is important, and the length of follow-up is impressive. However, the most interesting and novel questions appeared to yield null findings, which dampened enthusiasm overall. There are some additional concerns noted below.

Major Compulsory Revisions

1. Daughter's educational level is likely on the causal path (a putative mediator) between the exposures (maternal status/empowerment) and the outcomes (delay of sex, family planning, and unintended pregnancy). In order to truly extend the previous work, this mediational model should be considered.

2. Method: State how many dyads from the original sample were excluded (the original sample size is not noted) and whether the included dyads differed significantly from those excluded on key demographics and other variables.

3. Method: Clarify how the original contraception question was asked and what the response choices were. The method of dividing responses into "never" versus "ever" (the latter which would include sometimes and always) is inconsistent with some past research in the field. "Always" is often separated from all other categories (never and sometimes) because it only takes one unprotected sexual encounter

to result in an STI or an unintended pregnancy. The logic behind the subgroups needs to be conceptually justified and cited.

4. Method: The “well-kept” concept and the related item that resulted from qualitative work in this area (neat and tidy) seem conceptually linked to socioeconomic status or wealth rather than the other items that assessed empowerment (joint-decision making, economic control in the family). How correlated was the well-kept item with income, SES, or wealth? In general, the paper conflates the ideas of status and empowerment (and education), which all may be linked, but are conceptually different. Clarification and strong justification for why these concepts are all considered “empowerment” -- or alternatively, consideration of why they are unique -- is needed.

5. Method: The mother-daughter closeness and communication variables are interesting but testing them as covariates is not well-justified, as they do not precede the predictors. Consider testing them as mediators or effect modifiers (moderators) instead. This may provide more interesting or novel findings.

6. Results: A large percent of women reported never having intercourse at ages 25-26 (21% of the sample), which seems high. How does this compare to norms in this region (or to those excluded from this examination)? Raises concerns that the cohort or subsample is biased in some way, or women are inaccurately reporting. Same issue for the median age of sex (19 years).

7: Justify the inclusion of “withdrawal” as a family planning method.

8: Conclusion/discussion: It is unclear what this study adds to the literature given that previous studies with this sample have documented the association between maternal status and delay of sex. Generally, it appears that the questions that were most novel yielded null findings. As such, the present study seems incremental. Although 2 new outcomes are added, those associations were not significant.

9. The discussion overstates the study’s findings by suggesting that women’s empowerment is an important predictor of delaying sexual onset. The independent variables that appeared to be associated with this outcome were education, social status, etc. Labeling all as “empowerment” (and deemphasizing the lack of findings with the empowerment items) is not well-justified.

Minor Essential Revisions

10. The abstract would benefit from including a list of the covariates that were adjusted for in analyses.

11. In the intro, second paragraph from the last (which begins “In studies focusing on women’s empowerment...”) make clear that these studies were conducted in the Philippines. Clarify how the current study adds to the literature given findings from citations 31 and 32.

12. Method: it is unclear why the empowerment exposure is listed under a covariates subheader. Clarify which variables are covariates and which are exposure (predictor) variables.

13. The second sentence of the results section mixes the terms mean and median.

Discretionary Revisions (not for publication)

14. Results section of abstract: change “women’s empowerment among the mothers” which is unnecessarily wordy to maternal empowerment or mothers’ empowerment.

Level of interest - An article whose findings are important to those with closely related research interests

Quality of written English - Acceptable

I declare that I have no competing interests.

Response to reviewers

UNIVERSITY OF CALIFORNIA, LOS ANGELES **UCLA**

BERKELEY - DAVIS - IRVINE - LOS ANGELES - MERCED - RIVERSIDE - SAN DIEGO - SAN FRANCISCO SANTA BARBARA - SANTA CRUZ

SCHOOL OF PUBLIC HEALTH

P.O. BOX 951772
LOS ANGELES, CALIFORNIA 90095-1772

14 September 2016

Guest Editors
BMC Pregnancy & Childbirth

Dear BMC Pregnancy & Childbirth Editorial Committee,

On behalf of our research team, please find the attached, revised manuscript "Does mother's status and empowerment predict daughters' reproductive outcomes?" for consideration for publication in *BMC Pregnancy & Childbirth*. We appreciate the input from the reviewers. We provide our responses below and hope that we have adequately addressed their concerns.

In this manuscript, we use data from a unique, intergenerational and longitudinal dataset to examine the potential intergenerational effects of women's status and empowerment on subsequent reproductive outcomes in their daughters.

We find that that while some of the mother's characteristics and measures of empowerment and status were predictive of daughter's sexual initiation, these effects were not consistent across empowerment indicators, nor were there significant effects on two of the outcomes - use of family planning or occurrence of an unintended pregnancy. Older mothers (45+ years in 1994) and mothers who were considered to be 'well-kept', a locally-defined measure of empowerment, were more likely to have daughters who had not engaged in sex by 2009 (ages 25-26). Daughters with higher educational levels were also more likely to delay sex, as compared to their peers. Among young women who had become sexually active, 54% reported an unintended pregnancy (mistimed or unwanted) by the age of 25-26, yet their mother's empowerment and status were not predictive of daughters' reports of an unintended pregnancy.

Overall, these findings suggest that further research is needed to explore more proximal impacts on young women's reproductive behavior in this setting given other related investigations on women's empowerment and its linkages to sexual debut and educational attainment in this setting. Findings suggest that there are likely additional intervening mechanisms between onset on sexual activity and mistimed or unintended pregnancy that need further elaboration.

We hope you enjoy this revised manuscript and look forward to receiving your feedback.

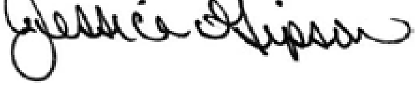

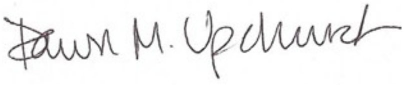

Sincerely,

Jessica Gipson, MPH PhD
Associate Professor
Department of Community Health Sciences

Dawn Upchurch, PhD L.Ac
Professor
Department of Community Health Sciences

Reviewer 1:
REVIEWERS REPORT

This is a beautifully written paper with a well conducted analysis that explores a relevant topic within a unique dataset, and will make an important contribution to the field. There are a few outstanding issues that the authors should address before the paper is ready for publication.

Major Compulsory Revisions

1. The empowerment "exposure variables" along with the daughters measures of closeness/communication were abstracted from the 1994 and 1998 surveys, which were collected 15 and 11 years (respectively) prior to the outcome measures. While there is an argument to be made that the mothers level of empowerment at that time has a predictive effect on the outcomes of interest, there is another argument to be made that empowerment levels, feelings of closeness, and even some of the basic socio-demographic characteristics that are included as covariates may change over time and those changed levels of exposures/covariates of interest (closer in time to the measured outcomes) may have more of an impact on the outcomes of interest than the levels measured in 1994/1998. It is possible that the authors do not have access to these same covariates at any other point in time, in which case they should discuss the relative merits and challenges with that lack of data and likely include that discussion in the limitations section. Or they may have access to such data and have made a conceptual decision not to include time varying covariates in the analysis. Either way, the authors must engage in a discussion of why the current conceptual model that measures levels of empowerment/closeness/communication in 1994/1998 is a reasonable model with which to predict the outcomes of interest 11-15 years later without accounting for changes in the exposure variables.

In the Introduction, we have further clarified that our conceptual model and subsequent analytic approach draw on aspects of lifecourse theory (page 4), in that we examine the potentially enduring influences of family of origin characteristics on daughter's reproductive behavior. We also tested the mediational effects of daughter's education (also to respond to Reviewer #2). Our empirical findings do show that these effects are enduring; however, only for initiation of sex. In our discussion, we discuss the potential influence and need for subsequent examination of more proximal influences, including the daughter's relationship partner and characteristics. To examine all of these potential pathways, however, is not within the scope of a single manuscript; we hope to build on this analysis in the future. We believe the manuscript, as revised, provides a new contribution to this literature.

2. Paragraph 2 of the Results section. It would be very helpful to see these data presented in tabular form.

We have included these data in tabular form (see Table 2).

3. Paragraph 4 of the Results section (beginning: Table 2…) needs to describe the difference between the adjusted and unadjusted models. What was controlled for exactly in the adjusted models?

Text has been added to Paragraph 4 in Results section.

4. Paragraph 5 of the Results section, lines 1-5, belong in the methods section, and I would like to see a more robust explanation of the theoretical construction of the model (this inevitably relates to comment #1)

See response below. We dropped the stepwise model building based on the Reviewer's concern. Our analytic strategy is now much more aligned with our theoretical groundings for this paper, as described further in the Introduction.

5. Paragraph 5 of the results section: I am not sure that you gain anything from the inclusion of the backwards stepwise model in the text. The "data driven model" approach can be useful at times, but given that you have some issues of selectivity in your sample, and may be missing some time varying factors, I would encourage you to stick with the conceptual/theoretically driven model as it has stronger a priori grounding and doesn't rely on data (which might be biased) to drive the model.

We agree and have deleted this paragraph from the Results section.

6. Paragraph 4 of the discussion section: you note that the rate of unintended pregnancy is alarmingly high in this population, but that is combining mistimed (75% of the sample) and unwanted pregnancies (15% of the sample). It seems like a bit more nuanced discussion of the potential differences between the two is warranted.

We appreciate the reviewer's note and agree that more explanation is needed. Please see revised text on page 8.

Reviewer 2:

I appreciated the opportunity to read this manuscript titled "Does mothers' status and empowerment predict daughters' reproductive outcomes?" which examined associations between maternal status/empowerment and daughters' sexual and reproductive outcomes. This study is interesting in its examination of intergenerational transmission of maternal status to reproductive outcomes in the next generation of girls and women using longitudinal data from the Philippines. Results showed that higher maternal status and education (not necessarily empowerment) was associated with one outcome, delayed sex among daughters, but not the other outcomes, contraceptive use or unintended pregnancy. The manuscript is well-written, the area of investigation is important, and the length of follow-up is impressive. However, the most interesting and novel questions appeared to yield null findings, which dampened enthusiasm overall. There are some additional concerns noted below.

Major Compulsory Revisions

1. Daughter's educational level is likely on the causal path (a putative mediator) between the exposures (maternal status/empowerment) and the outcomes (delay of sex, family planning, and unintended pregnancy). In order to truly extend the previous work, this mediational model should be considered.

We agree that this pathway is important to examine. In our preliminary runs of the data, we explored and tested for the role of daughter's education as a mediator in these pathways. However, in a comparison of models with and without daughter's education, there are no substantial differences in the effects sizes or significance of key variables indicating persistent, direct effects of the mother's status and empowerment variables. In addition, we have further developed our conceptual framework in the Introduction (see comments above) and specifically highlight that we also tested for mediation of daughter's education. Last, we have added some text in the Discussion (Results?) section describing these additional analyses that were performed.

2. Method: State how many dyads from the original sample were excluded (the original sample size is not noted) and whether the included dyads differed significantly from those excluded on key demographics and other variables.

The CLHNS dataset necessitates merging multiple modules per participant per data collection point; thus, dyads were constructed as a second step with the merged mother and daughter data. Therefore, comparing an 'original' sample of dyads is not possible. Several decisions along the way (e.g., excluding mothers who were not married/cohabiting in 1994) and limiting the sample to include only those mothers/daughters who persisted and provided data in subsequent rounds certainly presents some selectivity relative to the larger sample and population, as noted in the limitations section of the Discussion.

3. Method: Clarify how the original contraception question was asked and what the response choices were. The method of dividing responses into "never" versus "ever" (the latter which would include sometimes and always) is inconsistent with some past research in the field. "Always" is often separated from all other categories (never and sometimes) because it only takes one unprotected sexual encounter to result in an STI or an unintended pregnancy. The logic behind the subgroups needs to be conceptually justified and cited.

This is an unfortunate data limitation. Participants are only asked to report on 'ever use of family planning'. We also ran a model looking at modern contraceptive use as the outcome, though the overall findings did not differ.

4. Method: The "well-kept" concept and the related item that resulted from qualitative work in this area (neat and tidy) seem conceptually linked to socioeconomic status or wealth rather than the other items that assessed empowerment (joint-decision making, economic control in the family). How correlated was the well-kept item with income, SES, or wealth? In general, the paper conflates the ideas of status and empowerment (and education), which all may be linked, but are conceptually

different. Clarification and strong justification for why these concepts are all considered "empowerment" -- or alternatively, consideration of why they are unique -- is needed.
As noted in the manuscript and in the related citations, this measure of women's status was developed through extensive qualitative data collection in Cebu in the 1990's. The measure has subsequently been tested in several investigations, finding independent and significant effects, above and beyond those related to SES or wealth. In this dataset, the 'well-kept' measure is not highly correlated with SES/wealth (0.39), nor mother's education (0.30), and demonstrated independent, significant effects even in the multivariable models. This 'well-kept' measure in this and other analyses (cited in paper) appears to be capturing an additional dimension of women's well-being and empowerment that is not captured by parity, SES, wealth, or any of the additional proxy measures we used in analysis (and have been used broadly in the field) (e.g., age and educational differences, household decision-making, etc.).

5. Method: The mother-daughter closeness and communication variables are interesting but testing them as covariates is not well-justified, as they do not precede the predictors. Consider testing them as mediators or effect modifiers (moderators) instead. This may provide more interesting or novel findings.

These measures are included as a means of capturing the context of the mother-daughter relationship and in which the potential influences of the mother's status and empowerment may have been transmitted. These measures were not collected in the 1994 survey. To further explore the potential influences of these variables, we compared models with and without these variables, but the findings did not differ. A log-likelihood test confirmed that these two variables did not add to the predictive power of the model; however, we kept them in the model based on their theoretical/conceptual relevance.

6. Results: A large percent of women reported never having intercourse at ages 25-26 (21% of the sample), which seems high. How does this compare to norms in this region (or to those excluded from this examination)? Raises concerns that the cohort or subsample is biased in some way, or women are inaccurately reporting. Same issue for the median age of sex (19 years).
The statistics from this sample mirror nationally-representative data from the DHS. The 2013 Philippines Demographic Health Survey reports that 18.1% of 25-29 year olds have never had sex. Median age at first sex among 25-49 year olds is 21.5 years, and 21.0 years among 25-29 year olds. There is likely inaccurate reporting of first sex among women in both the CLHNS and the DHS, given social stigma regarding premarital sex, particularly for women. It is not clear, however, that the CLHNS data are any less valid in this regard given the rapport that study interviewers have with participants.

8: Conclusion/discussion: It is unclear what this study adds to the literature given that previous studies with this sample have documented the association between maternal status and delay of sex. Generally, it appears that the questions that were most novel yielded null findings. As such, the

present study seems incremental. Although 2 new outcomes are added, those associations were not significant.

Respectfully, it is our belief that even null findings are a contribution to the literature. These findings can inform subsequent investigations of alternate pathways or ways of modeling these outcomes.

9. The discussion overstates the study's findings by suggesting that women's empowerment is an important predictor of delaying sexual onset. The independent variables that appeared to be associated with this outcome were education, social status, etc. Labeling all as "empowerment" (and deemphasizing the lack of findings with the empowerment items) is not well-justified.

Duly noted. The Abstract, as well as first and last paragraphs of the Discussion have been revised accordingly.

Other Revisions

10. The abstract would benefit from including a list of the covariates that were adjusted for in analyses. We have crafted the abstract to conform to the word limits, as well as the style of the journal. Readers may refer to tables and text to identify specifics of the modeling (including covariates).

11. In the intro, second paragraph from the last (which begins "In studies focusing on women's empowerment...") make clear that these studies were conducted in the Philippines. Clarify how the current study adds to the literature given findings from citations 31 and 32.

Edited.

12. Method: it is unclear why the empowerment exposure is listed under a covariates subheader. Clarify which variables are covariates and which are exposure (predictor) variables.

Edited.

13. The second sentence of the results section mixes the terms mean and median.

The text is correct as-is. We reported both the mean (19.61) and the median (19 years) here as an indication of the distribution of age at first sex.

14. Results section of abstract: change "women's empowerment among the mothers" which is unnecessarily wordy to maternal empowerment or mothers'

empowerment.

Edited.

Review reports – 2nd round

Reviewer 1 : Caitlin Gerdts

I am happy for it to move forward for publication as is.

Reviewer 2: Julianna Deardorff

I do not have additional comments. I am convinced by the authors' responses that, while the statistically significant findings are somewhat incremental, the publication of null findings is important. The revisions have resulted in a stronger paper overall and, at this point, I would recommend it for publication.
